# The Endocytic Adaptor Eps15 Controls Marginal Zone B Cell Numbers

**DOI:** 10.1371/journal.pone.0050818

**Published:** 2012-11-30

**Authors:** Benedetta Pozzi, Stefania Amodio, Caterina Lucano, Anna Sciullo, Simona Ronzoni, Daniela Castelletti, Thure Adler, Irina Treise, Ingrid Holmberg Betsholtz, Birgit Rathkolb, Dirk H. Busch, Eckhard Wolf, Helmut Fuchs, Valérie Gailus-Durner, Martin Hrabě de Angelis, Christer Betsholtz, Stefano Casola, Pier Paolo Di Fiore, Nina Offenhäuser

**Affiliations:** 1 IFOM Fondazione Istituto FIRC di Oncologia Molecolare, Milan, Italy; 2 IEO Istituto Europeo di Oncologia, Milan, Italy; 3 German Mouse Clinic, Institute of Experimental Genetics, Helmholtz Center, Munich-Neuherberg, Germany; 4 Institute for Medical Microbiology, Immunology and Hygiene, Technische Universität München, Munich, Germany; 5 Department of Medical Biochemistry and Biophysics, Karolinska Institute, Stockholm, Sweden; 6 Gene Center, Ludwig-Maximilians-Universität München, Munich, Germany; 7 Chair for Experimental Genetics, Technische Universität München, Freising-Weihenstephan, Germany; 8 University of Milan, Milan, Italy; University of Freiburg, Germany

## Abstract

Eps15 is an endocytic adaptor protein involved in clathrin and non-clathrin mediated endocytosis. In *Caenorhabditis elegans* and *Drosophila melanogaster* lack of Eps15 leads to defects in synaptic vesicle recycling and synapse formation. We generated Eps15-KO mice to investigate its function in mammals. Eps15-KO mice are born at the expected Mendelian ratio and are fertile. Using a large-scale phenotype screen covering more than 300 parameters correlated to human disease, we found that Eps15-KO mice did not show any sign of disease or neural deficits. Instead, altered blood parameters pointed to an immunological defect. By competitive bone marrow transplantation we demonstrated that Eps15-KO hematopoietic precursor cells were more efficient than the WT counterparts in repopulating B220^+^ bone marrow cells, CD19^−^ thymocytes and splenic marginal zone (MZ) B cells. Eps15-KO mice showed a 2-fold increase in MZ B cell numbers when compared with controls. Using reverse bone marrow transplantation, we found that Eps15 regulates MZ B cell numbers in a cell autonomous manner. FACS analysis showed that although MZ B cells were increased in Eps15-KO mice, transitional and pre-MZ B cell numbers were unaffected. The increase in MZ B cell numbers in Eps15 KO mice was not dependent on altered BCR signaling or Notch activity. In conclusion, in mammals, the endocytic adaptor protein Eps15 is a regulator of B-cell lymphopoiesis.

## Introduction

Marginal zone (MZ) B cells are mature B cells that reside in the sinus of the spleen. These cells are directly exposed to the circulating blood stream, and consequently, to blood-borne pathogens. MZ B cells, therefore, are ideally placed to mount a rapid, T cell-independent, IgM host response to blood-borne antigens. In addition, MZ B cells have a low threshold for activation by antigens. While this property is essential to guarantee a rapid host response, it also might also lead to higher auto-reactivity. As a consequence, hyperactivation of MZ B cells has been implicated in the pathogenesis of autoimmune diseases, such as systemic lupus erythematosus (SLE) [1], [2].

The development of MZ B cells is only partially understood. Immature B cells expressing a non-autoreactive B-cell receptor (BCR) are generated, throughout the lifetime of individuals, from progenitor B cells present in the bone marrow. These newly-generated B cells migrate into the blood stream and, after entering into the spleen, progress through two consecutive transitional B cell stages, T1 and T2 [3]. The T2 transitional B cell is thought to be the common precursor for both MZ B and follicular B-2 B cells [4]. Studies on gene-targeted mice have allowed the identification of several MZB cell determinants [5], [6], [7], [8], [9], [10], including effectors of Notch [11], [12], [13], [14], [15], [16], [17] and BCR [18], [19], although their contribution to MZB cell establishment/maintenance remains poorly understood [3], [20].

Endocytic control of receptors involved in immune function has been widely documented. In particular, the role of BCR internalization and trafficking has been extensively studied in the context of B cell activation following antigen binding [21]. Much less is known about how endocytosis might impinge on developmental decisions necessary to establish a functional immune system.

**Figure 1:**
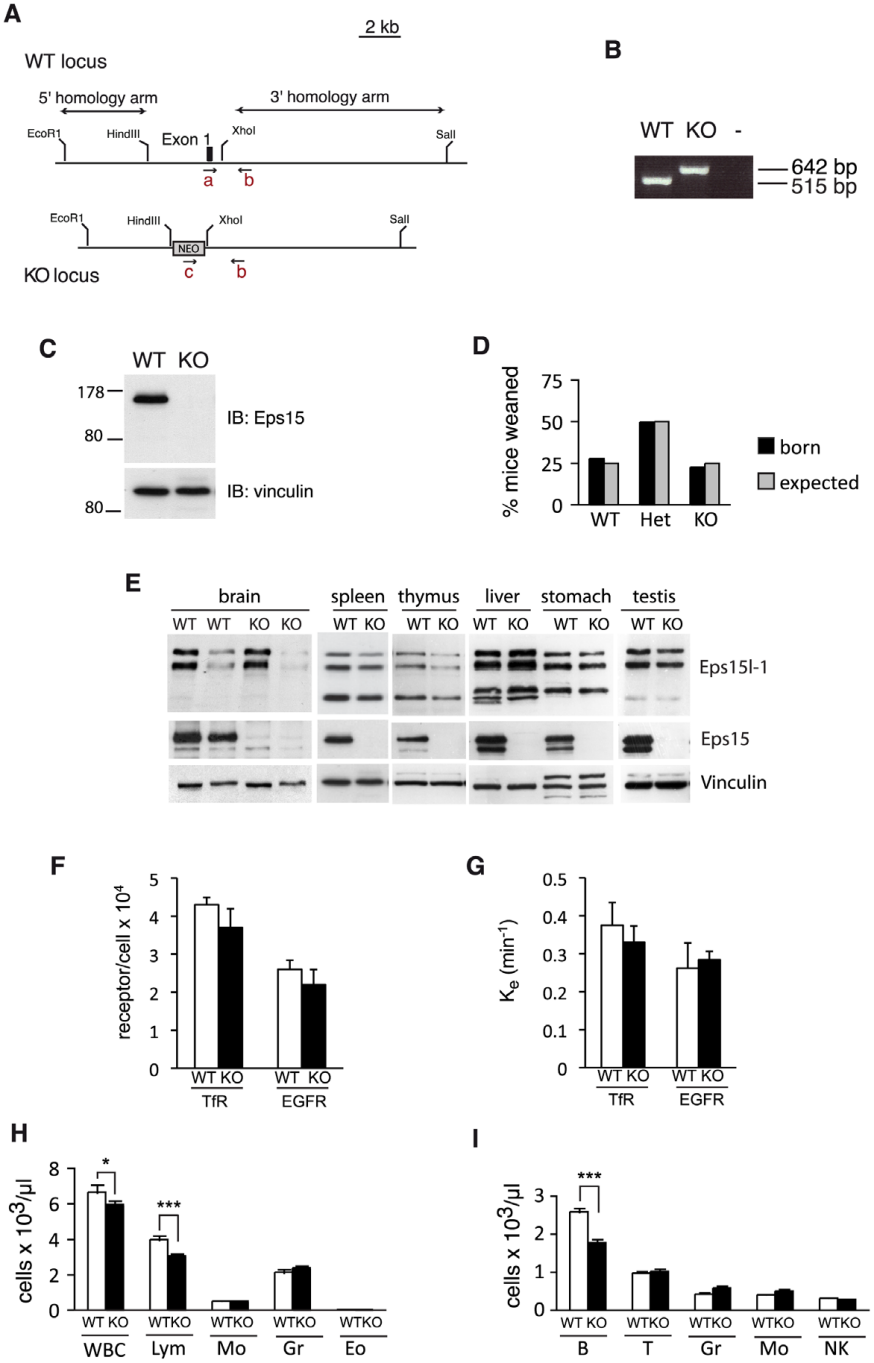
Eps15-KO mice are viable and show an immunological defect. A. Scheme depicts the region within the Eps15 wildtype (WT) locus that was altered to generate the knockout (KO) locus. The first coding exon of Eps15 and 2 kb of the 5′ promoter region were replaced by gene targeting with a neomycin (NEO) cassette. The location of the primers (a, b, c) used for genotyping is indicated (not to scale). **B.** Three primer PCR on tail biopsies gave the expected bands for the WT and KO allele: 515 and 642 bp, respectively. **C.** Western blot of total spleen lysates from Eps15-WT (WT) or Eps15-KO (KO) mice decorated with an anti-Eps15 antibody, or anti-vinculin antibody as loading control, shows lack of Eps15 protein in Eps15-KO mice. **D.** Bar graph depicting the percent of mice that have been weaned (black bars) for the indicated genotypes (wildtype (WT), heterozygous (Het), knockout (KO)) from the breeding of Eps15 heterozygous mice. The expected frequency for each genotype is depicted by the grey bars. A total of 231 pups were analyzed. **E.** Western blot analysis of multiple tissues from Eps15-WT (WT) and Eps15-KO (KO) mice reveals ubiquitous expression of Eps15 and Eps15L1. Total tissue lysates (10 µg protein) were separated by SDS-PAGE, transferred to nitrocellulose membrane and probed for Eps15, Eps15L1 or vinculin, as loading control, as indicated. For brain lysates obtained from Eps15-WT (WT) or Eps15-KO (KO) mice, 10 and 20 µg protein were loaded to allow a better comparison of Eps15 and Eps15L1 protein levels. **F.–G.** Bar graphs displaying cell surface receptor levels (**F**) and internalization rate constants Ke (**G**) for the transferrin receptor (TfR) and the EGF receptor (EGFR) in Eps15-WT (white bars) and Eps15-KO (black bars) primary mouse fibroblasts. **H.** Bar graph depicting the number of white blood cells (WBC), lymphocytes (Lym), monocytes (Mo), granulocytes (Gr) and eosinophils (Eo) in the peripheral blood of 3-month-old Eps15-WT (WT, white bars, n = 10) and Eps15-KO (KO, black bars, n = 10) mice as determined by hematological analysis. **I.** Bar graph depicting the number of B cells (B), T cells (T), granulocytes (Gr), monocytes (Mo) and natural killer cells (NK) in the peripheral blood of 3 month old Eps15-WT (WT, white bars, n = 10) and Eps15-KO (KO, black bars, n = 10) mice as determined by FACS analysis. Statistical significance was assessed using Student’s t-test. Significant differences are indicated as * = p<0.05, *** = p<0.005.

Eps15, originally cloned as a phosphorylated substrate of the EGFR, is an endocytic adaptor protein implicated both in clathrin- and non-clathrin mediated endocytosis [22], [23], [24], [25]. By virtue of its interaction with a number of diverse binding partners, such as Numb, Epsin, AP-2, Stonin, Parkin, and Ubiquilin [26], [27], [28], [29], [30], Eps15 is likely to be involved in a variety of cellular and biological processes. In *D. melanogaster* and *C. elegans*, similarly to other endocytic proteins [31], [32], [33], [34], [35], Eps15 is critically involved in synaptic vesicle recycling [36], [37], [38]. Additionally, in *D. melanogaster* loss of Eps15 leads to altered synapse formation and larval lethality [36], [37].

**Figure 2:**
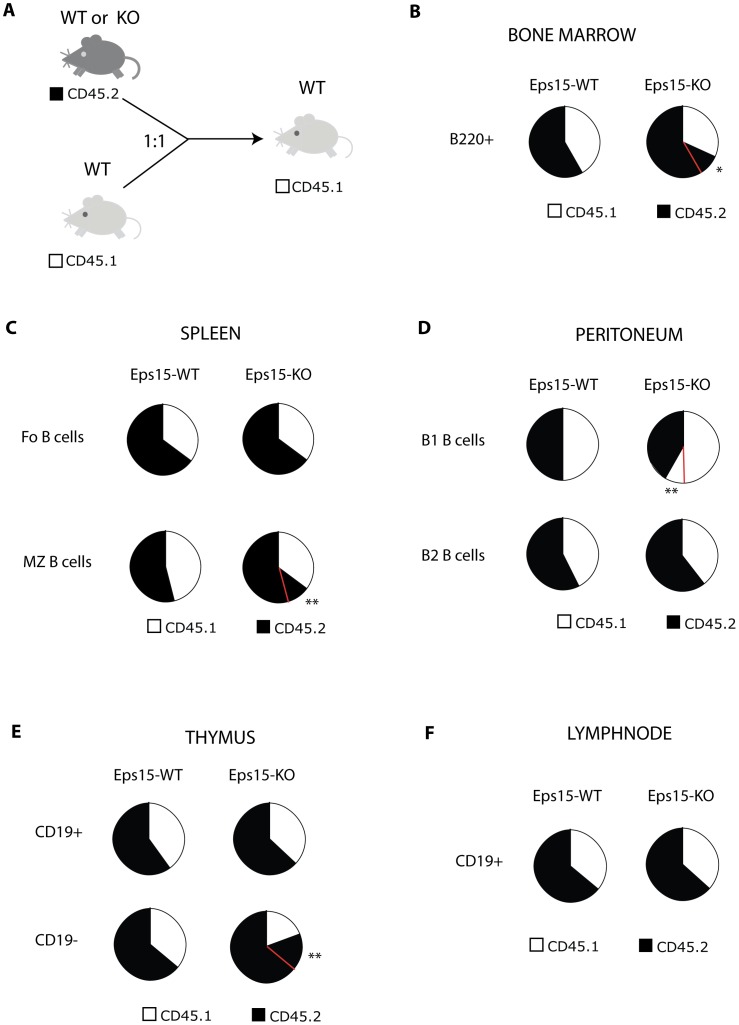
Eps15 has multiple roles in the immune system. **A.** Scheme depicting the outline of the competitive bone marrow transplantation experiment. Bone marrow cells from Eps15-WT or -KO mice were mixed with WT bone marrow cells at a 1∶1 ratio and injected into sub-lethally irradiated recipient mice. The origin of the cells in the recipient mice was traced taking advantage of isogenic variation of the CD45 locus: CD45.2 is present in Eps15-WT and Eps15-KO mice, while CD45.1 is present in the wildtype (WT) mice used as competitor donor and as recipient mice. **B.–F.** Pie charts depicting the relative contribution of CD45.2 (Eps15-WT or Eps15-KO) versus WT CD45.1 positive competitor cells in the bone marrow (**B**), spleen (**C**), peritoneum (**D**), thymus (**E**) and lymph nodes (**F**) of recipient mice, as determined by FACS analysis. Significant differences (*p<0.05, **p<0.01) in the competition between Eps15-WT (n = 4) and Eps15-KO (n = 5) CD45.2^+^ cells with CD45.1^+^ WT cells, are indicated by red lines in the pie charts.

**Table 1:** FACS analysis of the B cell populations present in the bone marrow three months after competitive bone marrow transplantation.

Donor	1∶1	WT:WT		1∶1	WT:KO	
% cells	gated	CD45.1+	CD45.2+	gated	CD45.1+	CD45.2+
FSC-A/SSC-A	24.1	29.5	37.4	28.2	23.9	46.4***
B220+	53.9	39.9	56.1	58.5	30.6*	66.0*
AA4.1+	47.6	37.9	57.7	51.3	26.1	70.6
IgM-CD23+	1.3	48.7	47.1	2.2	28.7*	66.9*
IgM+CD23+	6.3	46.1	47.3	7.1	30.7*	62.9*
IgM+CD23−	16.9	38.4	57.6	18.4	25.6	70.8
IgM-CD23−	75.4	36.9	56.8	72.3	25.8	69.2
FSC-A/SSC-A	61	31.1	50.3	55.0	22.9*	56.4
B220-	72.3	23.7	39.2	66.1	17.2	39.6
B220+IgM+	23.5	42.3	54.9	24.8	29.4	62.7
B220++IgM+	76.1	42.3	54.9	74.2	33.6*	63.1*
B220+IgM−	10.8	42.7	48.0	12.8	27.0*	57
Pre B	11.2	46.4	49.4	10.4	13.4***	53.7
Pro B	82.0	42.3	50.0	84.8	28.4	56.4

Distribution of CD45.1+ and CD45.2+ cells in the bone marrow of recipient mice 3 months after bone marrow transplantation. CD45.1+ WT and CD45.2+ WT or CD45.2+ KO donor cells were mixed at a 1∶1 ratio prior to injection into recipient CD45.1+ mice. The percentage of total cells gated and the percentage of CD45.1+ or CD45.2+ cells for any given gate are shown. Significance was assessed using Student’s t-test and p-values are indicated as. *p<0.05, **p<0.01, ***p<0.001.

During evolution, many endocytic proteins underwent gene duplication and, whereas *D. melanogaster* and *C. elegans* often possess only one copy of a given gene, mammals frequently evolved two or three functional paralogs. Eps15 is a case in point, with two closely related genes in both mice and humans, namely, Eps15 and its homolog, Eps15L1. Genetic duplication has permitted the endocytic network to become more robust, so that genetic deletion of any one member in mice, in general, has no or relatively mild effects [39], [40], [41]. An additional consequence of gene duplication is the acquisition of a new function(s) by one or other of the paralogous genes.

**Table 2:** FACS analysis of the cellular populations present in the spleen three months after competitive bone marrow transplantation.

Donor	1∶1	WT:WT		1∶1	WT:KO	
% cells	gated	CD45.1+	CD45.2+	gated	CD45.1+	CD45.2+
FSC-A/SSC-A	82.5	39.7	58.2	82.5	37.1	61.0
CD19+	68.5	35.2	62.9	66.4	33.7	64.3
Fo	81.9	35.4	63.8	81.1	35.1	64.0
MZ	6.2	44.7	52.9	5.5	35.14*	62.54**
CD1d+/T1	3.4	23.1	60.9	3.8	19.4	65.3
Cd1d+/T2-Fo	91.1	35.5	63.5	89.8	34.7	63.9
CD19+CD1d++	4.2	40.3	56.3	4.0	33.16*	63.86*
MZ	61.7	44.4	52.8	62.2	33.24***	64.38***
Pre-MZ	9.6	33.5	59.1	6.6	37.7	54.5
FSC-A/SSC-A	81.7	39.9	58.6	80.34	37.52	61.1
B220+	69.3	35.6	63.1	67.3	34.0	64.6
AA4.1+	13	34.7	62.3	14	25.3*	71.9*
IgM-CD23+	42.6	38.7	59.5	40.8	28.2*	70.2*
IgM+CD23+	26.3	34.6	62.4	25.3	24.1**	73.3**
IgM+CD23−	18.6	29.9	65.3	20.0	20.8**	73.8*
IgM−CD23−	12.5	28.8	66.7	14	24.7	72.2
FSC−A/SSC-A	82.6	40.1	58.5	82.3	37.4	61.1
CD19−	30.4	50.4	47.4	34.1	43.7	54.4
CD8−CD4+	56.9	50.6	49.3	59.9	46	53.8
CD8+CD4+	0.2	47.6	45.1	0.2	42.5	47
CD8+CD4−	29.1	53.7	45.8	25.7*	46.5	53.1
CD8−CD4−	13.9	42.2	43.7	14.2	29.8*	58.6*
FSC-A/SSC-A	91.8	35.9	58.5	91.8	34.1	61.4
CD11c+	4.4	35.7	51.4	4.6	26.0*	62.2*
Gr1+	15.4	51.0	33.9	13.3	42.9	43.7
Mac1+	17.0	39.8	39.1	15.8	27.6	53.9

Distribution of CD45.1+ and CD45.2+ cells in the spleen of recipient mice 3 months after bone marrow transplantation. CD45.1+ WT and CD45.2+ WT or CD45.2+ KO donor cells were mixed at a 1∶1 ratio prior to injection into recipient CD45.1+ mice. The percentage of total cells gated and the percentage of CD45.1+ or CD45.2+ cells for any given gate are shown. Significance was assessed using Student’s t-test and p-values are indicated as *p<0.05, **p<0.01, ***p<0.001.

To define the function of Eps15 in a mammalian organism, we generated Eps15 knockout (Eps15-KO) mice. Eps15-KO mice are viable and fertile, which allowed us to perform an extensive phenotypic analysis. We report here an unexpected role of Eps15 in the immune system. We found that Eps15-KO mice show increased MZ B cell numbers. This phenotype is cell autonomous and Notch independent. Competitive bone marrow transplantation revealed a preferential reconstitution of thymic and bone marrow cells by Eps15-KO hematopoietic precursors, suggesting that multiple signaling pathways, impinging on diverse developmental decisions, are controlled by Eps15.

**Table 3:** FACS analysis of the cellular populations present in the thymus three months after competitive bone marrow transplantation.

Donor	1∶1	WT:WT		1∶1	WT:KO	
% cells	gated	CD45.1+	CD45.2+	gated	CD45.1+	CD45.2+
FSC-A/SSC-A	95.5	35.7	63.6	95.5	19.4**	79.9**
CD19+	0.4	38.1	56.5	0.5	33.7	58.3
CD19−	99.5	35.8	63.7	99.5	19.4**	80.0**
CD8−CD4+	9.2	39.4	60.5	10.9	21.4**	78.4**
CD8+CD4+	86.3	35.4	64.3	84.2	19.0**	80.5**
CD8+CD4−	2.6	41.8	57.8	3.2	22.4**	77*
CD8−CD4−	1.9	19.4	45.9	1.7	17.4	69.3

Distribution of CD45.1+ and CD45.2+ cells in the thymus of recipient mice 3 months after bone marrow transplantation. CD45.1+ WT and CD45.2+ WT or CD45.2+ KO donor cells were mixed at a 1∶1 ratio prior to injection into recipient CD45.1+ mice. The percentage of total cells gated and the percentage of CD45.1+ or CD45.2+ cells for any given gate are shown. Significance was assessed using Student’s t-test and p-values are indicated as *p<0.05, **p<0.01, ***p<0.001.

## Results

Eps15-KO mice were generated by deleting the first coding exon harboring the first ATG codon and replacing it with a neomycin resistance gene (Figure 1A). We used PCR to confirm correct gene targeting (Figure 1B) and western blotting to verify the loss of Eps15 protein in Eps15-KO mice (Figure 1C). Eps15-KO mice were born at the expected Mendelian ratio (Figure 1D) and were healthy and fertile, with no obvious phenotypes. Western blot analysis of multiple tissues revealed that Eps15 and Eps15L1 have similar expression patterns in different tissues and are ubiquitously expressed (Figure 1E). In addition, in Eps15-KO mice, Eps15L1 levels were comparable to those in Eps15-WT mice, indicating that there was no compensation for the lack of Eps15 by Eps15L1 (Figure 1E). The absence of Eps15 did not affect transferrin or EGF cell surface receptor levels (Figure 1F). Ligand-induced internalization of the EGFR, as well as constitutive internalization of the transferrin receptor (Figure 1G) were not altered in Eps15-KO compared with Eps15-WT fibroblasts, suggesting that the role of Eps15 in internalization of these receptors is compensated by other proteins.

**Table 4:** FACS analysis of the B cell populations present in the peritoneal cavity three months after competitive bone marrow transplantation.

Donor	1∶1	WT:WT		1∶1	WT:KO	
% cells	gated	CD45.1+	CD45.2+	gated	CD45.1+	CD45.2+
FSC-A/SSC-A	56.2	46.4	52.4	43.1	41.5	53.5
B1	11.1	50.1	48.6	9.2	63.4**	34.8**
CD5+	57.2	47.3	51.3	52.4	60.9*	37.5*
CD5−	41.4	53.2	45.9	46.2	66.1***	32.1***
B2	57.2	42.1	56.7	53.2	38.8*	59.9
CD5+	16.6	38.7	58.2	13.7	38.2	58.0
CD5−	81.0	42.9	56.4	83.3	39.0*	60.1

Distribution of CD45.1+ and CD45.2+ cells in the peritoneal cavity of recipient mice 3 months after bone marrow transplantation. CD45.1+ WT and CD45.2+ WT or CD45.2+ KO donor cells were mixed at a 1∶1 ratio prior to injection into recipient CD45.1+ mice. The percentage of total cells gated and the percentage of CD45.1+ or CD45.2+ cells for any given gate are shown. Significance was assessed using Student’s t-test and p-values are indicated as *p<0.05, **p<0.01, ***p<0.001.

In order to uncover any disease-related phenotypes in Eps15-KO mice, we performed a comprehensive screen including more than 300 parameters to assess neurological, behavioral, cardiovascular, metabolic and immunologic function (the complete screen can be found at http://www.mouseclinic.de/). At variance with the established role of Eps15 in synaptic vesicle recycling and synapse development in *D. melanogaster* and *C. elegans* [36], [37], [38], Eps15-KO mice did not show alterations related to the nervous system. This lack of overt neurological defects in Eps15-KO mice is likely due to functional redundancy with the related protein, Eps15L1, as will be discussed subsequently.

**Table 5:** FACS analysis of the B cell populations present in the lymph nodes three months after competitive bone marrow transplantation.

Donor	1∶1	WT:WT		1∶1	WT:KO	
% cells	gated	CD45.1+	CD45.2+	gated	CD45.1+	CD45.2+
FSC-A/SSC-A	77.5	43.5	55.4	79.0	40.5	58.5
CD19+	53.9	35.4	62.1	43.7	36.2	61.8
IgM-IgD+	84.3	36.6	62.4	87.6*	35.6	63.5
IgM+IgD+	7.8	35.7	62.7	7.4	36.2	61.9
IgM+IgD−	0.3	31.8	62.6	0.3	28.1	62.5
IgM−IgD−	7.6	55.1	41.6	4.7*	43.2	53.2
Ungated cells		40.8	52.5		37.9	55.0
CD19+	49.0	37.6	59.3	38.2**	35.8	61.7
CD38+	90.8	35.9	62.1	94.9**	35.7	62.5
FAS+	6.3	65.2	32.7	2.9*	47.7	48.7
GC	1.4	23.1	40.2	0.9*	23.2	47.0
FSC-A/SSC-A	84.3	43.9	55.1	86.6	40.7	58.2
CD19+	46.4	50.3	48.3	60.2*	43.7	54.9
CD8−CD4+	59.8	50.4	49.0	60.9*	44.3	55.3
CD8+CD4+	0.3	35.6	38.6	0.2	33.8	41.3
CD8+CD4−	33.8	52.6	47.1	33.1	45.2	54.7
CD8−CD4−	6.2	38.0	47.9	5.7	28.7	53.5

Distribution of CD45.1+ and CD45.2+ cells in the lymph node of recipient mice 3 months after bone marrow transplantation. CD45.1+ WT and CD45.2+ WT or CD45.2+ KO donor cells were mixed at a 1∶1 ratio prior to injection into recipient CD45.1+ mice. The percentage of total cells gated and the percentage of CD45.1+ or CD45.2+ cells for any given gate are shown. Significance was assessed using Student’s t-test and p-values are indicated as *p<0.05, **p<0.01, ***p<0.001.

The only significant and reproducible alteration monitored in Eps15-KO mice resulted from the analysis of hematological parameters (http://www.mouseclinic.de/). Notably in Eps15-KO mice, the percentage of lymphocytes was reduced (Figure 1H), whereas that of other blood cells was comparable to control animals. We confirmed this finding by FACS analysis of peripheral blood (Figure 1I), which established a primary defect in B-, but not T-lymphocytes.

**Table 6:** FACS analysis of the B cell populations present in the Peyer’s patches three months after competitive bone marrow transplantation.

Donor	1∶1	WT:WT		1∶1	WT:KO	
% cells	gated	CD45.1+	CD45.2+	gated	CD45.1+	CD45.2+
Ungated cells		20.1	32.3		24.4	33.5
CD19+	45.3	28.5	53.6	46.5	34.9	50.7
CD38+	68.8	32.9	63.0	72.0	38.8	57.1*
FAS+	5.2	50.0	38.3	5.3	50.5	40.1
GC	22.9	10.6	25.5	19.7	12.9	28.0

Distribution of CD45.1+ and CD45.2+ cells in the Peyer’s patches of recipient mice 3 months after bone marrow transplantation. CD45.1+ WT and CD45.2+ WT or CD45.2+ KO donor cells were mixed at a 1∶1 ratio prior to injection into recipient CD45.1+ mice. The percentage of total cells gated and the percentage of CD45.1+ or CD45.2+ cells for any given gate are shown. Significance was assessed using Student’s t-test and p-values are indicated as *p<0.05, **p<0.01, ***p<0.001.

The lack of Eps15 leads, therefore, to a decrease in circulating B cells. The fact that most parameters were normal and that Eps15-KO were healthy and fertile excludes major hematological defects in the mutant mice. Functional impairments linked to gene inactivation are best assessed under competitive conditions. Therefore, we decided to perform a competitive bone marrow transplantation (BMT) assay in which mutant cells compete with their wildtype (WT) counterparts for repopulation of the hematopoietic lineages. Bone marrow cells from Eps15-WT or Eps15-KO mice were mixed with WT bone marrow cells at a 1∶1 ratio and injected into sub-lethally irradiated recipient WT mice (Figure 2A). The fate of the transplanted cells was followed by FACS analysis, taking advantage of the congenic marker CD45, with CD45.2 being carried by Eps15-WT or -KO mice, and CD45.1 being instead expressed by the WT competitor cells and recipient mice. Thus, the relative contribution of Eps15-WT or -KO cells, with respect to competitor WT cells, was determined by comparing the proportion of CD45.2^+^ versus CD45.1^+^ cells in any given cellular subset. Using this strategy, we observed an increased contribution of Eps15-KO cells compared with Eps15-WT cells to bone marrow B220^+^ cells (Figure 2B, Table 1), splenic MZ, but to not Fo, B cells (Figure 2C, Table 2), and to CD19^−^ thymocytes (Figure 2E, Table 3); Eps15-KO cells instead contributed less efficiently to peritoneal B1 B cells (Figure 2D, Table 4). Comparable reconstitution efficiency between wild-type and mutant cells was seen in the pool of CD19^+^ B-cells of peripheral lymph nodes (Figure 2F, Table 5) and Peyer’s patches (Table 6).

**Figure 3:**
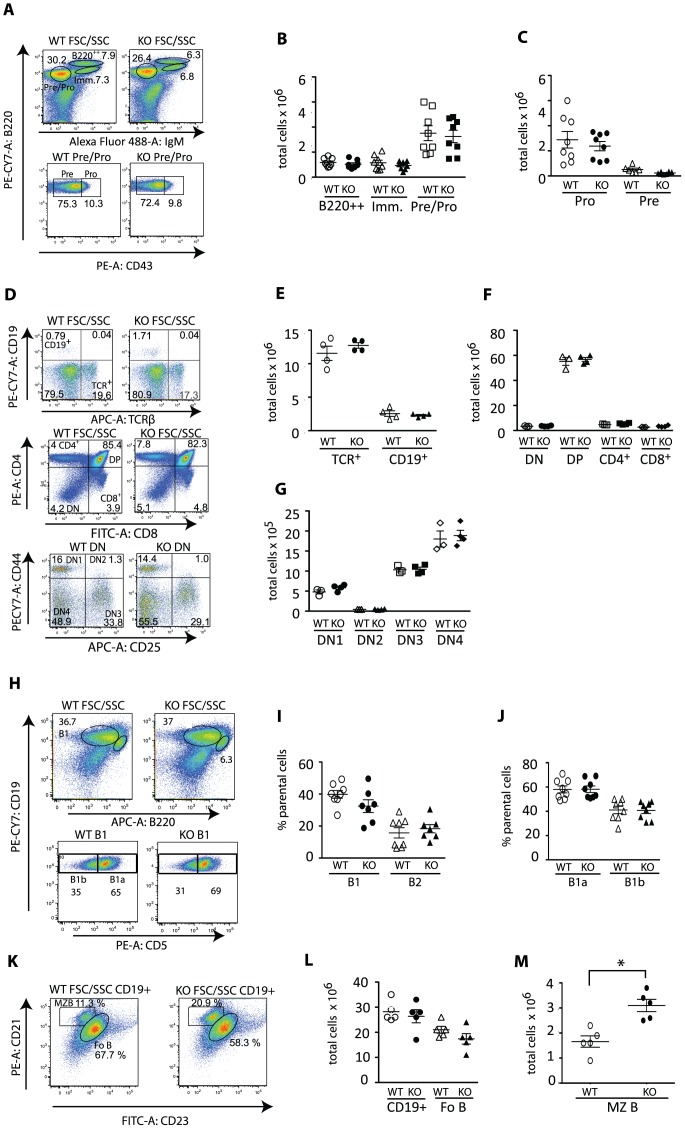
Eps15-KO mice show increased marginal zone B cell numbers. **A.** Dot plots of bone marrow cells from 4-month-old Eps15-WT and Eps15-KO mice stained for B220 and IgM to identify pre−/pro-B cells (B220^+^IgM^−^), immature (B220^+^IgM^+^) and mature (B220^high^IgM^+^) B cells. Pre−/pro-B cells were further analyzed for expression of CD43 to identify pro- (CD43^+^) and pre- (CD43^−^) B cells. **B.–C.** Dot plots depicting the total number of mature (B220^++^), immature (Imm.) and pre−/pro- and B cells (**B**) and pre- and pro-B cells (**C**) in the bone marrow of Eps15-WT (WT, white symbols, n = 5) and Eps15-KO (KO, black symbols, n = 6) mice. **D.** Top panel: dot plot of thymocytes from 2-month Eps15-WT and Eps15-KO mice stained for CD19 and TCRβ to identify B and T cells, respectively. Middle panel: dot plots for thymocytes from 4-month-old Eps15-WT and Eps15-KO mice staind for CD4 and CD8 to identify thymocyte subpopulations. Bottom panel: the CD4/CD8 double negative population (DN) was further analyzed for immature thymocytes using CD44 and CD25. **E.–G.** Dot plots depicting the total number of the following cell populations in the thymi of Eps15-WT (WT, white symbols) and Eps15-KO (KO, black symbols) mice: (**E**) TCRβ^+^ and CD19^+^ cells (n = 3), (**F**) DN (double negative, CD4^−^CD8^−^), DP (double positive, CD4^+^CD8^+^), CD4^+^ and CD8^+^ (n = 4) and (**G**) of DN1 (CD44^+^CD25^−^), DN2 (CD44^+^CD25^+^), DN3 (CD44^−^CD25^−^) cells (n = 4). **H**. Dot plot of peritoneal B cells from 2–4 month old Eps15-WT and Eps15-KO mice stained for CD19 and B220 gated for CD19^+^B220^low^ B1 and CD19^+^B220^high^ B2 B cells. B1 B cells were further stained and gated for CD5 to identify CD5^+^ B1a and CD5^−^ B1b B cells. **I.–J.** Dot plots depicting the percentage of B1 and B2 (I) and B1a and B1b (J) B cells in the peritoneum of Eps15-WT (WT, white symbols) and Eps15-KO (KO, black symbols) mice (n = 8). **K.** Dot plot of splenocytes from Eps15-WT and Eps15-KO mice stained for CD19 and gated for CD21 and CD23 to identify Fo B (CD19^+^/CD21^+^/CD23^+^) and MZ B (CD19^+^/CD21^+^/CD23^−^) cells. **L.** Dot plots depicting the total number of CD19+, Fo B and MZ B cells in the spleens of Eps15-WT (WT, white symbols) and Eps15-KO (KO, black symbols) mice (n = 5). Values are depicted as mean±standard error mean. Statistical significance was assessed using Student’s t-test and significant differences are indicated as * = p<0.05.

To understand whether these alterations in lymphocyte differentiation were already present at steady state in Eps15-KO mice, we analyzed the bone marrow, thymus, peritoneum and spleen from control and mutant mice. We observed normal numbers of B cells and B cell progenitors in the bone marrow of Eps15-KO mice (Figure 3A–C). Similarly, we observed normal B and T cell numbers in the thymus of Eps15-KO mice (Figure 3D–G), as well as a normal distribution of peritoneal B cells (Figure 3H–J). By contrast, we noted a two-fold average increase in MZ B cells in the spleen of Eps15-KO mice compared to WT mice, while total B and Fo B cell numbers were unaffected in mutant mice (Figure 3 K–M). Thus, the preferential contribution of Eps15-KO cells to the MZ B cell compartment seen in the competitive BMT assays is mirrored by an increase in MZ B cell numbers in Eps15-KO mice.

**Figure 4:**
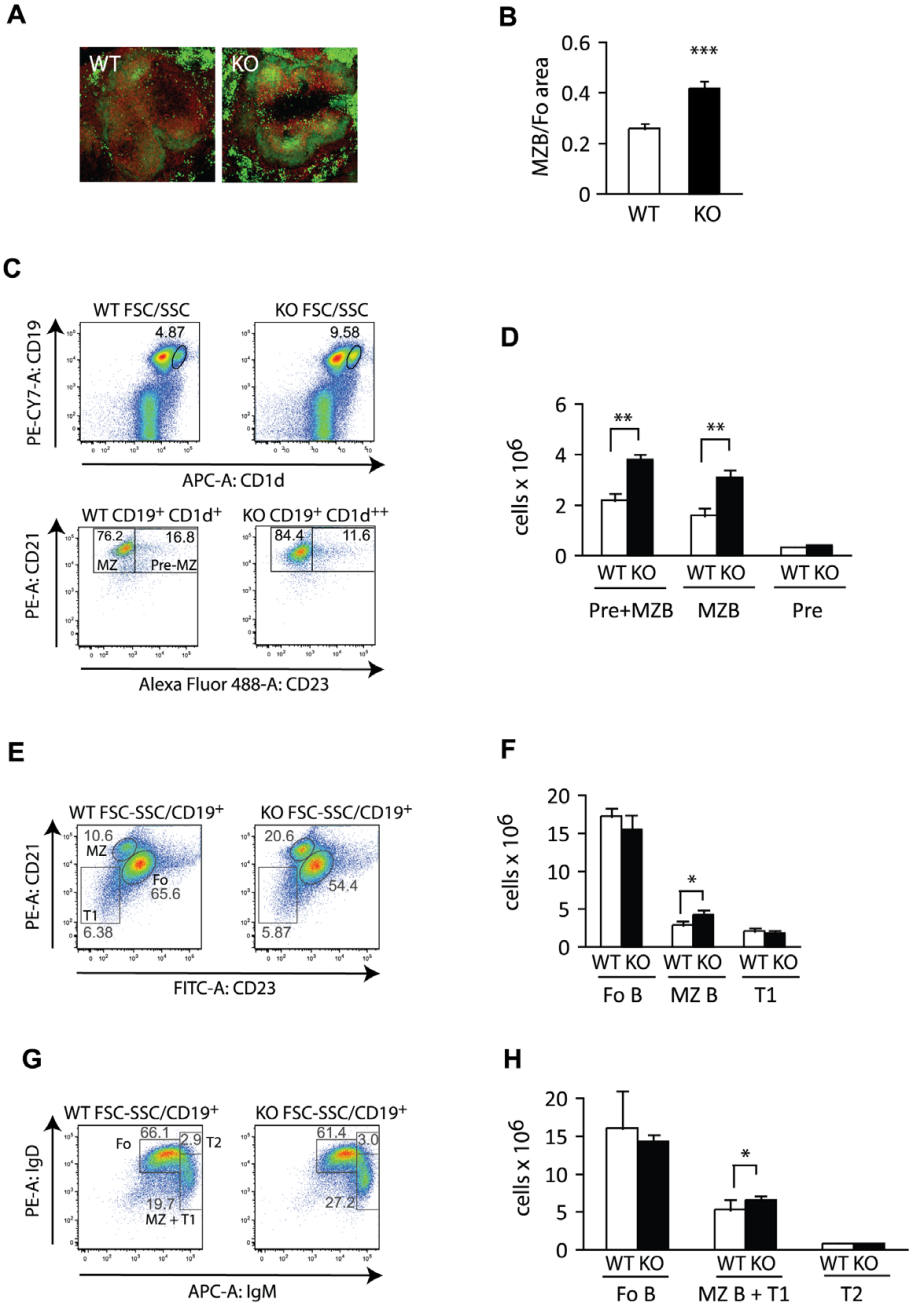
Eps15-KO mice show normal pre-MZ B and transitional B cell numbers. A. Photomicrographs of spleen sections from adult Eps15-WT and Eps15-KO mice. Sections were stained for IgD (PE, red) and IgM (FITC, green) to visualize MZ B (IgM^+^IgD^low/−^) and Fo B cells (IgD^+^IgM^−^). **B.** Bar graph representing the quantification of the ratio of the area occupied by MZ B cells over the area occupied by Fo B cells in Eps15-WT and Eps15-KO spleens (n = 12 per genotype). **C.** Dot plots of splenocytes from Eps15-WT and Eps15-KO mice stained for CD19 and CD1d to identify the pre-MZ B plus MZ B (pre+MZB) cell populations. This mixed CD19^+^CD1d^++^ population was further analyzed for CD23 to distinguish pre-MZ B (CD19^+^/CD1d^++^/CD23^+^) from mature MZ B (CD19^+^/CD1d^++^/CD23^−^) cells. **D.** Bar graph depicting total cell numbers of pre+MZB cells, mature MZ B and pre-MZ B cells in the spleens of Eps15-WT (WT, white bars) and Eps15-KO (KO, black bars) mice (n = 5). **E.** Dot plots of splenocytes from Eps15-WT and Eps15-KO mice stained for CD19, CD21 and CD23 to identify Fo B (CD19^+^/CD21^+^/CD23^+^), MZ B (CD19^+^/CD21^+^/CD23^−^) and T1 transitional B (CD19^+^/CD21^−/^CD23^−^) cells. **F.** Bar graph depicting total cell numbers of Fo B, MZ B and T1 transitional B cells in the spleens of Eps15-WT (WT, white bars) and Eps15-KO (KO, black bars) mice (n = 5). **G.** Dot plots of splenocytes from Eps15-WT and Eps15-KO mice stained for CD19, IgD and IgM to identify Fo B (CD19^+^/IgD+IgM^Low^), MZ B plus T1 transitional B (MZ B+T1, CD19^+^/IgD^low/−^IgM^+^) and T2 transitional B (CD19^+^/IgD^+^IgM^+^) cells. **H.** Bar graph depicting total cell numbers of Fo B, MZ B+T1, and T2 transitional B cells in the spleens of Eps15-WT (WT, white bars) and Eps15-KO (KO, black bars) mice (n = 5). Values are depicted as mean±standard error mean. Statistical significance was assessed using Student’s t-test and significant differences are indicated as *p<0.05, **p<0.01, ***p<0.005.

To confirm the increase in the size of the MZ B cell pool, we performed an immunofluorescence analysis on spleen sections from WT and Eps15-KO mice. The thickness of the cellular rim surrounding the marginal sinus consisting of IgM^+^IgD^−^ MZ B cells was increased in mutant animals when compared with controls. (Figure 4A). Statistical analysis of the data demonstrated a significant increase in the MZ/Fo area in Eps15-KO respect to WT mice (Fig. 4B). MZ B cells develop in the spleen where they progress through the T1 and T2 transitional stages before becoming mature MZ B cells [42]. To understand whether the loss of Eps15 impinges on any of the early stages of MZ B cell development, we analyzed precursor and mature MZ B cell populations in Eps15-KO mice by flow cytometry. As shown in Figure 4 C–D, while we could validate the increase in the fraction of MZ B cells using two independent marker combinations (Cd19^+^CD1d and IgM^+^IgD^low/−^), we did not see any alterations in pre-MZ B, T1 or T2 transitional B cell numbers in Eps15-KO mice (Figure 4 C–H). Likewise, there was no difference in Fo B cell numbers (Figures 4E–H). Thus, the increase in mature MZ B cells cannot be attributed to changes in the number of precursor cell populations.

**Figure 5:**
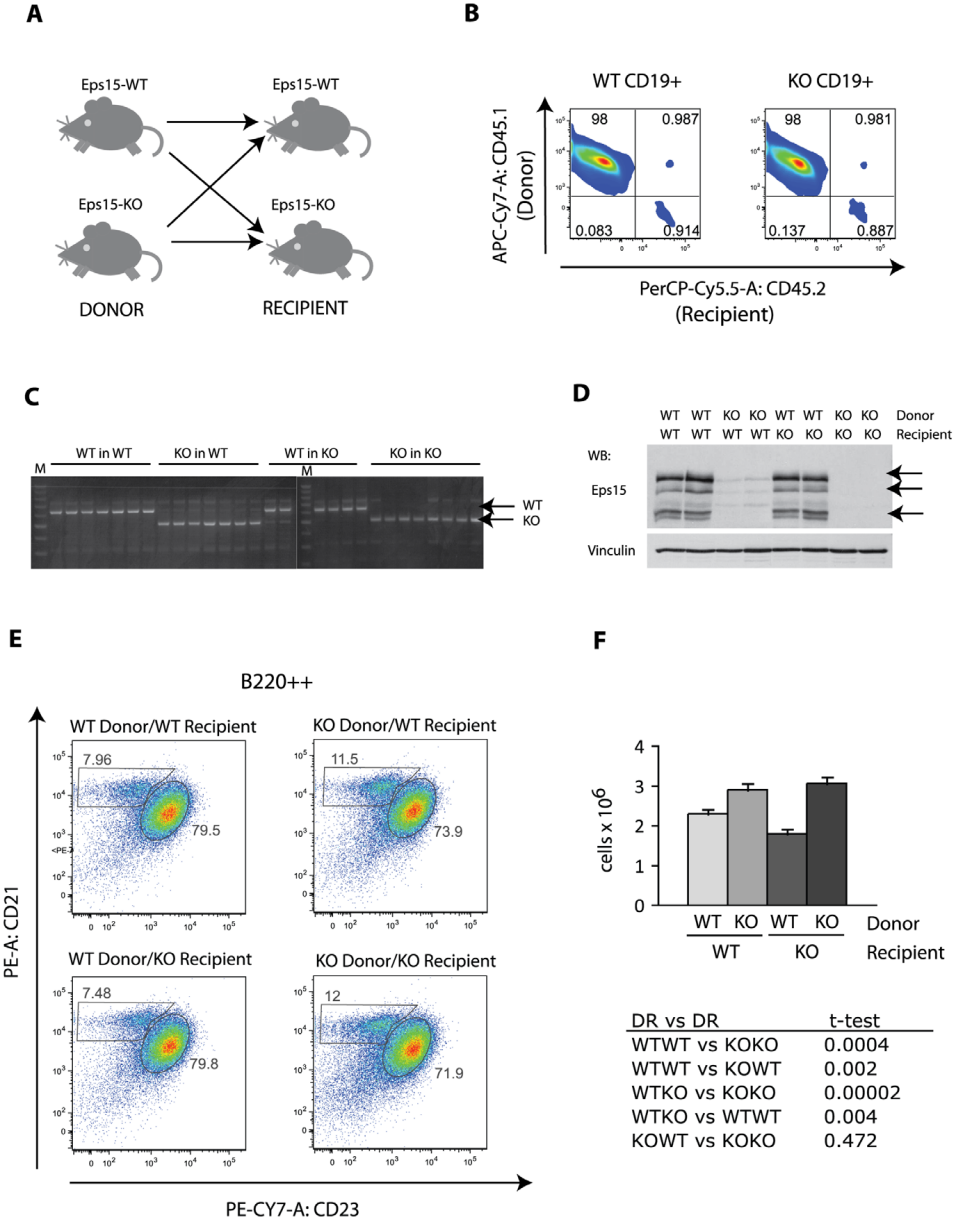
The increase in the MZB cell population in Eps15-KO mice is cell autonomous. A. Scheme depicting the experimental design of the reverse bone marrow transplantation. Bone marrow cells from Eps15-WT or Eps15-KO mice were transplanted into sub-lethally irradiated Eps15-WT or Eps15-KO mice. **B.** Dot plot depicting the efficiency of CD45.1^+^ WT donor cell engraftment in either Eps15-WT or Eps15-KO CD45.2^+^ recipient mice. Gating CD19^+^ splenocytes for CD45.1 and CD45.2 expression revealed more than 99% engraftment of donor cells in both types of host. **C.** PCR genotyping of peripheral blood 2 months after bone marrow transplantation. In mice injected with Eps15-WT cells the resulting genotype is WT, independent of the recipient genotype. Similarly, in mice injected with Eps15-KO cells the resulting genotype is KO, independent of the recipient genotype. ‘M’ indicates marker. **D.** Western blot analysis of splenocyte lysates 3 months after bone marrow transplantation. The membrane was decorated with anti-Eps15, or anti-vinculin as a loading control. The Eps15-specific bands are indicated by arrows. **E.** Dot plot of splenocytes 3 months after bone marrow transplantation. Splenocytes were stained for CD19, CD21 and CD23 to identify Fo B (CD19^+^/CD21^+^/CD23^+^) and MZ B (CD19^+^/CD21^+^/CD23^−^) cells for each combination of genotypes. **F.** Bar graph depicting the total number of CD19^+^/CD21^+^/CD23^−^ MZ B cells for each genotype combination (n = 6). The significance was calculated using the Student’s t-test and is reported in the table below the graph.

To understand whether the increase in MZ B number is B cell autonomous we performed a reverse BMT. Bone marrow cells from either Eps15-WT or -KO mice were injected into sub-lethally irradiated Eps15-WT or -KO recipient mice (Figure 5A). To follow the fate of the injected cells, we first tested whether the engraftment into Eps15-KO mice was as efficient as into Eps15-WT mice. To do so we injected CD45.1^+^ WT cells into Eps15 control and mutant recipients. In both cases, the injected cells contributed to over 99% of the splenocytes, of the host (Figure 5B). In support of this result, the genotype of blood cells of reconstituted mice corresponded to that of the donor cells (Figure 5C) and western blot analysis on protein extracts from splenocytes of control and mutant recipients confirmed the presence or absence of Eps15 protein (Figure 5D). We then proceeded to analyze the number of MZ B cells in the four combinations of reconstituted animals and found that there was a significant increase in MZ B cells when the donor cells were derived from Eps15-KO mice, regardless of the genotype of the recipients (Figure 5E, F). This result indicates that the increase in the size of the MZ B cell population observed in Eps15-KO mice is cell autonomous.

**Figure 6:**
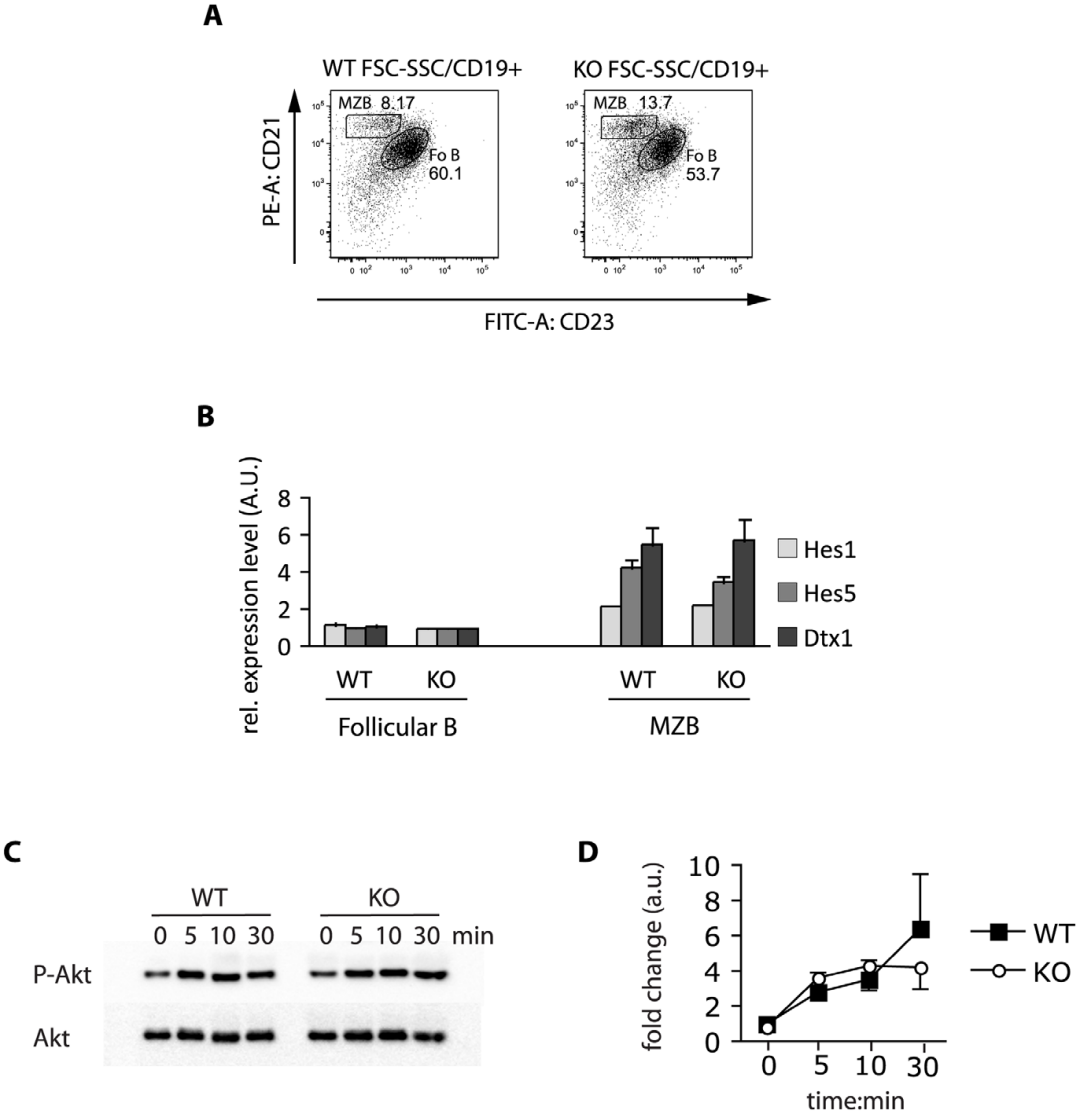
Notch target gene expression and BCR signaling is comparable in MZ B cells from Eps15-WT and Eps15-KO mice. A. Dot plot depicting the sorting scheme used to purify Fo B (CD19^+^/CD21^+^/CD23^+^) and MZ B (CD19^+^/CD21^+^/CD23^-^) cells from Eps15-WT and Eps15-KO mice. **B.** Bar graph depicting the relative expression levels of the indicated Notch target genes (Hes1, light grey bar; Hes5, grey bar; Deltex (Dtx1), dark grey bar) in Fo B and MZ B cells from Eps15-WT (WT) or Eps15-KO (KO) mice (n = 6). **C.** Western blot analysis of purified splenic B-cells, obtained from 2-month-old Eps15-WT or Eps15-KO mice, after stimulation with 5 ug/ml anti-IgM for 0, 5, 10 or 30 minutes. Lysates were probed for phospho-Akt (P-Akt) or Akt for normalization, as indicated. **D.** Line graph displaying the fold-change of Phospho-Akt/Akt as detected by western blotting of splenic B cells from Eps15-WT (WT, black squares) and Eps15-KO (KO, white circles) after stimulation with anti-IgM as reported in **C**. Values are derived from three independent experiments and are expressed in arbitrary units (a.u.).

Notch signaling plays a key role in the regulation of MZ B cell development [13], [14], [16], [43]. Indeed, knockout mice impaired in Notch signaling show decreased MZ B cell numbers [11], [44]. Thus, to determine whether Notch signaling is responsible for the increased MZ B cell number in Eps15-KO mice, we sorted Fo B and MZ B cells from Eps15-WT and -KO mice (Figure 6A) and analyzed the expression of Notch target genes by QPCR. Expression of Hes1, Hes5 or Deltex in both Fo B and MZ B cells was comparable between Eps15-WT and -KO mice (Figure 6B), suggesting that the MZ B cell phenotype in Eps15-KO mice is independent of Notch signaling. BCR signaling has also been shown to influence MZ B cell development [18], [19], [45]. We thus tested BCR activation in Eps15 null cells by stimulating purified splenic B cells *in vitro* with anti-IgM, using Akt activation as a read-out. No difference was observed in P-Akt levels, suggesting that BCR signaling is not affected in Eps15-KO mice (Figure 6C–D).

**Figure 7:**
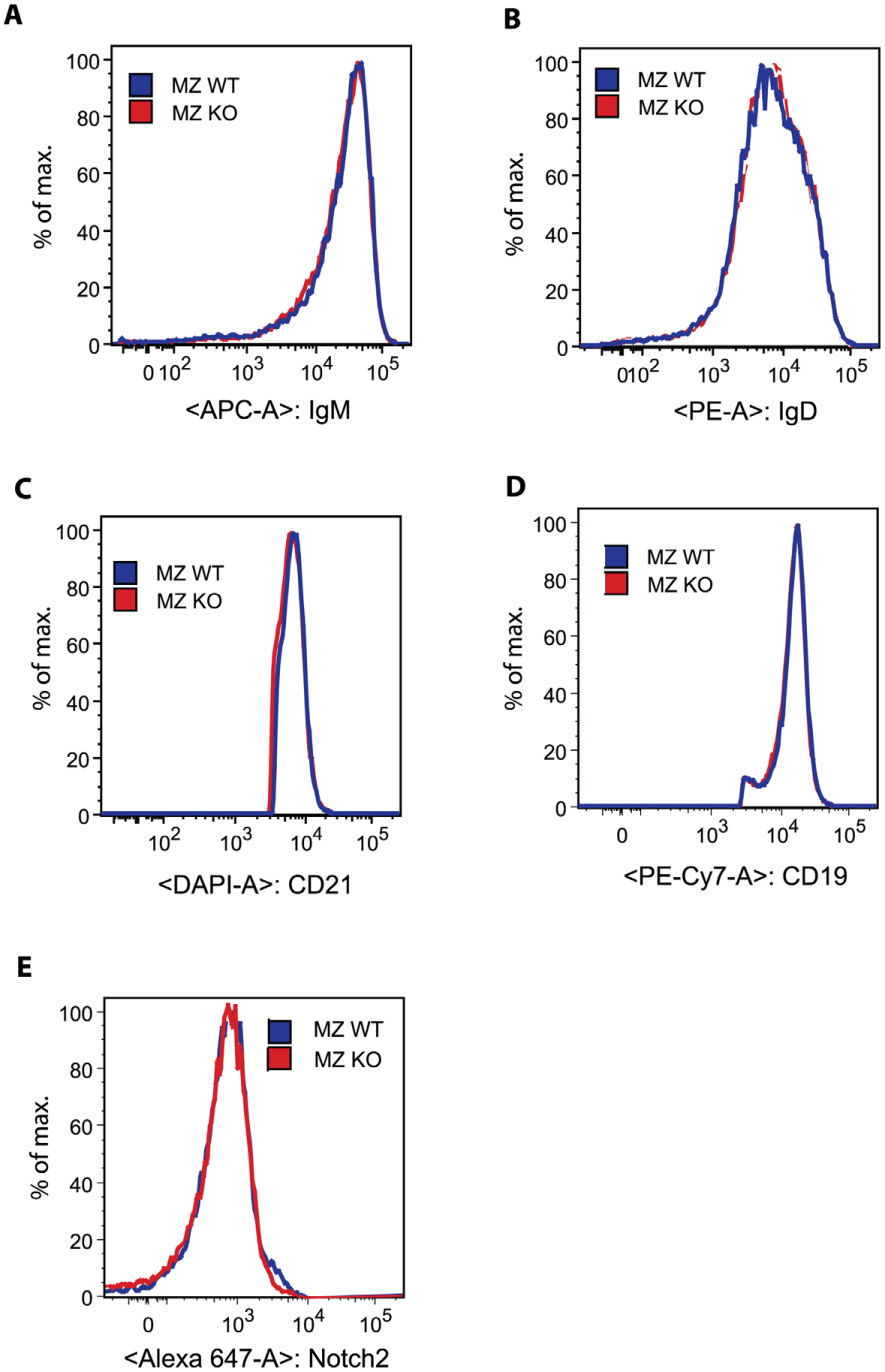
Normal levels of cell surface receptors in Eps15-KO MZ B cells. Representative histograms from MZ B cells from 4-month-old Eps15-WT (blue trace) and Eps15-KO (red trace) mice stained for IgM-APC (**A**), IgD-PE (**B**), CD21-DAPI (**C**), CD19-PE-CY7 (**D**) and Notch2 (**E**).

Given that Eps15 is an endocytic adaptor protein, its absence might impinge on receptor steady-state levels. Instead, surface levels of IgM, IgD, CD21 and CD19 were not altered in Eps15-KO mice (Figure 7A–D). Similarly, Notch2 surface levels were normal when compared to WT (Figure 7E), in line with the finding that Notch target genes were not altered in Eps15-KO MZ B cells.

**Figure 8:**
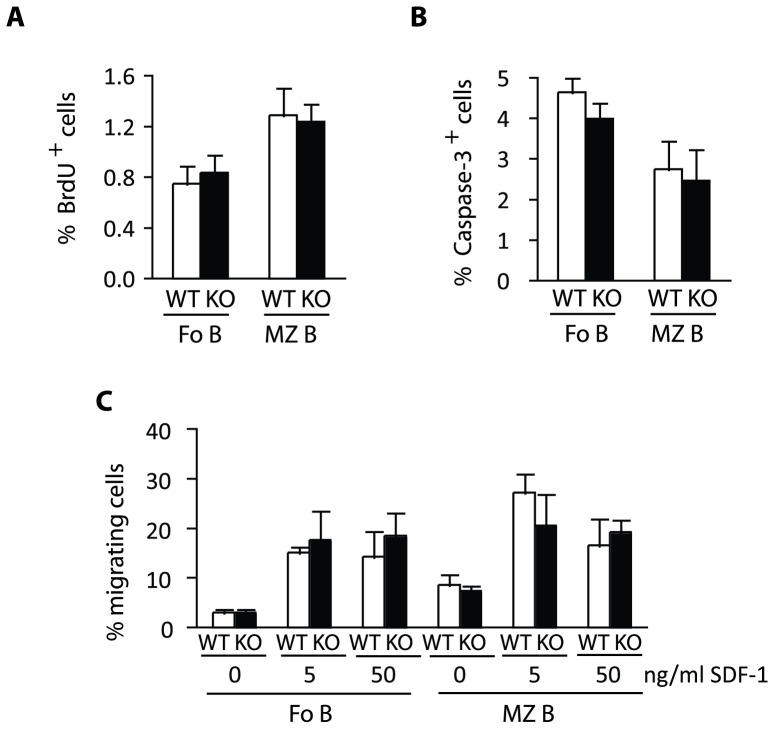
Normal proliferation and apoptosis levels in Eps15-KO MZ B cells. A.–B. Bar graph depicting the percentage of BrdU-positive (BrdU+) (**A**) or active caspase-3-positive (Caspase+) (**B**) in Fo and MZ B cells in 2−4-month-old Eps15-WT (WT, white bars) and Eps15-KO (KO, black bars) mice 4 hrs after a BrdU pulse (n = 8). **C.** Bar graph depicting the percentage of Fo and MZ B cells migrating towards increasing concentrations of the chemokine SDF-1 in a transwell assay using purified splenic B cells from 3-month-old Eps15-WT (WT, white bars) and Eps15-KO (KO, black bars) mice (n = 4).

Increased MZ B cell numbers could result from increased proliferation and/or reduced apoptosis, however no difference in either proliferation or apoptosis was observed in Eps15-KO MZ or Fo B cells (Figure 8A, B). We next assessed whether altered migration could account for increased MZ B cell numbers. Transwell migration assay towards stromal derived factor 1 (SDF-1), failed to detect differences in the chemotoxis of Eps15-KO B cells (Figure 8C).

**Figure 9:**
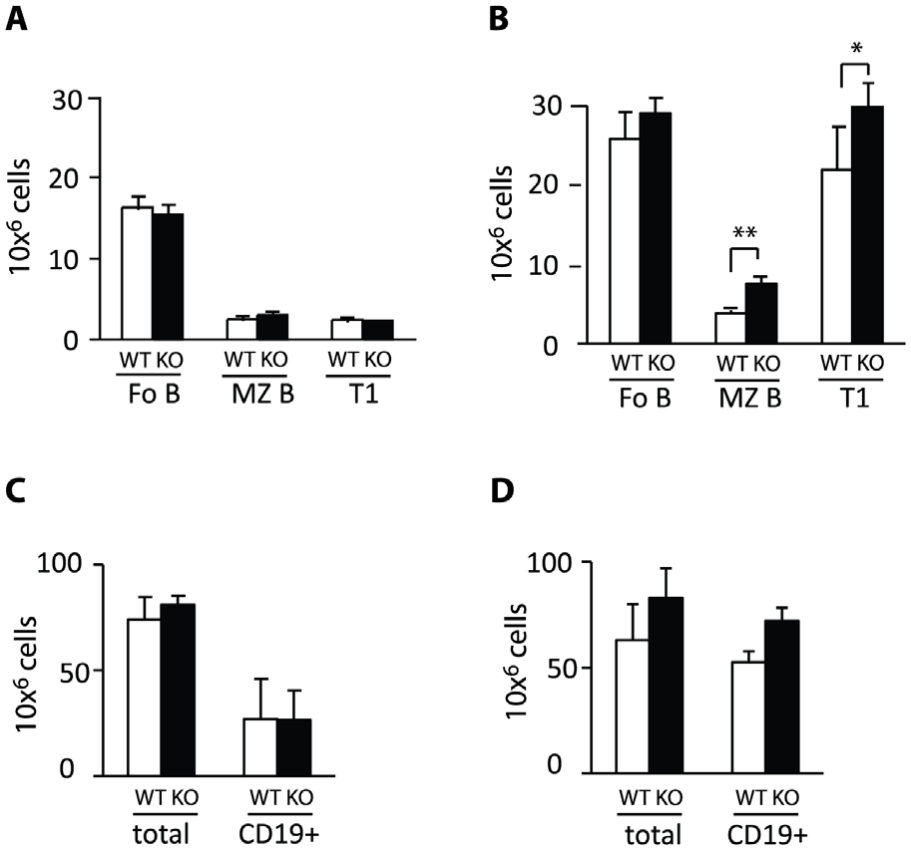
The MZ B cell numbers increase over time in Eps15-KO mice. A.–B. Bar graphs depicting the total number of Fo, MZ and T1 B cells in the spleen of 2 month old (**A**) and 24 month old (**B**) Eps15-WT (WT, white bars) and Eps15-KO (KO, black bars) mice. **C.–D.** Bar graphs depicting the total and CD19+ cell number in the spleen of 2-month-old (**A**) and 24-month-old (**B**) (Eps15-WT (WT, white bars) and Eps15-KO (KO, black bars) mice. Values are depicted as mean±standard error mean. N = 4 per two month old and n = 5 per 24 month old mice. Statistical significance was assessed using Student’s t-test and significant differences are indicated as * = p<0.05, ** = p<0.01.

Our inability to detect mechanistic or functional differences that lead to the increase in MZ B cell numbers in Eps15-KO mice, could be explained with a slow continuous accumulation of the cells over time. We thus assessed whether the phenotype was detectable already in young mice. Two-months-old Eps15-KO mice, in contrast to 4-month-old mice (Figure 4), did not display any increase in MZ B cells compared with Eps15-WT mice (Figure 9A). At 24 months of age, however Eps15-KO mice displayed a marked increase in MZ and additionally in T1 B cells (Figure 9B). Thus as anticipated, MZ B cells increase over time in Eps15-KO mice while total splenic B cell numbers are not altered (Figure 9C, D).

**Table 7:** Normal Ig and absence of auto-antibodies in young Eps15-KO mice.

	Male (n = 15, each)		Female (n = 15, each)	
	Eps15-WT	Eps15-KO	Eps15-WT	Eps15-KO
IgG1	167.3±16.4	191.4±17.8	130.1±13.0	162.3±14.0
IgG2a	83.3±14.0	70.0±8.1	64.2±7.5	54.8±7.9
IgG2b	171.5±29.2	246.1±28.5	284.3±35.4	398.2±45.7
IgG3	99.1±15.2	170.9±45.1	145.7±18.8	96.8±20.2
IgM	1002.0±203.7	886.5±171.3	784.4±29.2	722.7±115.6
IgA	336.5±148.4	234.3±47.1	150.0±20.5	134.4±20.7
Anti-DNA Ab	0	0	0	0
Rheumatoid factor	0	0	0	0

Antibody titers were determined in serum from 12-week old mice.

**Figure 10:**
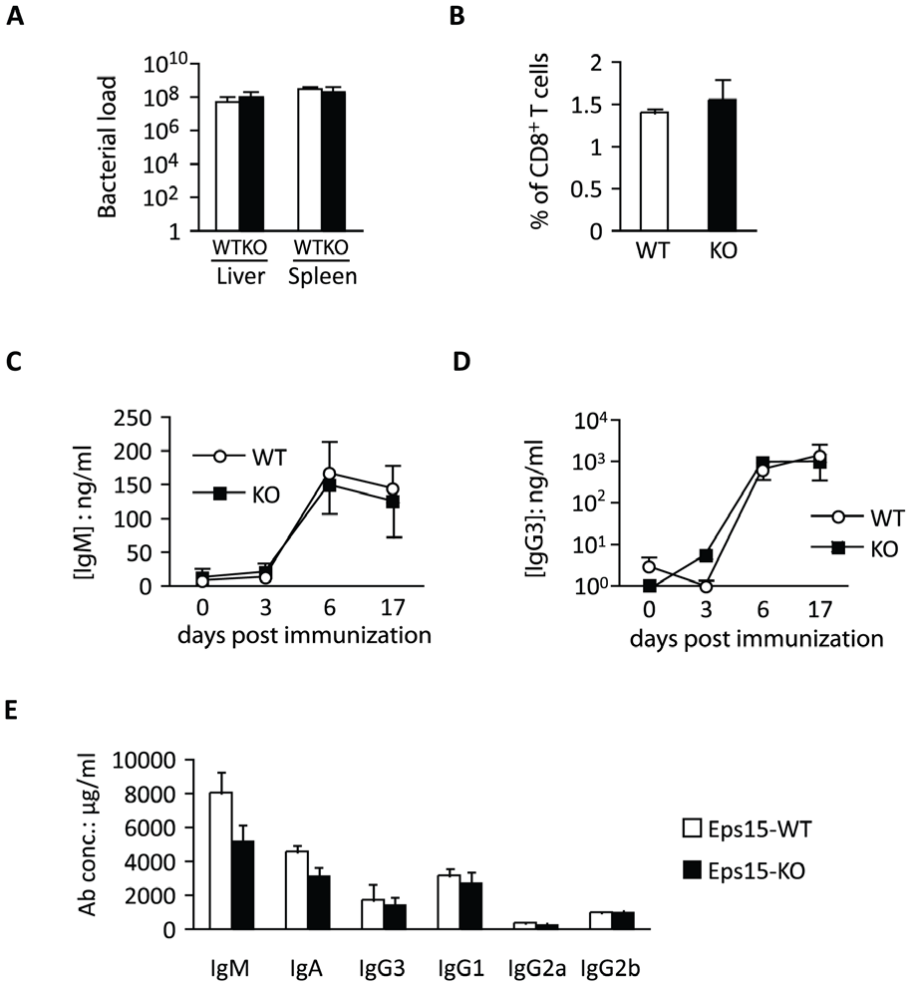
Normal immune-reactivity in Eps15-KO mice. A. Bar graph depicting the bacterial load of Listeria monocytogenes in the liver and spleen of Eps15-WT (WT, white bar) and Eps15-KO (KO, black bar) 3 month old mice (n = 6), three days post-infection. **B.** Bar graph depicting the percentage of Listeria responsive CD8^+^ splenic T cells in 3 month old Eps15-WT (WT, white bar, n = 2) and Eps15-KO (KO, black bar, n = 3) mice seven days post-infection. **C.**–**D.** Line graph depicting the concentration of IgM (**C.**) and IgG3 (**D.**) in the serum of 2 month old Eps15-WT (WT, white circle, n = 5) and Eps15-KO (KO, black square, n = 6) mice before immunization and 3, 6 and 17 days post-immunization with 5 µg NP-Ficoll. **E.** Bar graph depicting the concentration of antibodies in the blood of 24-month-old Eps15-WT (WT, white bar, n = 8) and Eps15-KO (KO, black bar, n = 11) mice.

We next addressed whether the lack of Eps15 might lead to detectable alterations in the immune function of MZ B cells. We assessed lymphocyte reactivity by quantifying steady state levels of serum Ig (Table 7), the immune response to the pathogen Listeria (Figure 10A, B) and to the T-cell independent antigen NP-Ficoll (Figure 10 C, D). No differences were detected between Eps15-KO and WT mice. To evaluate auto-reactivity, we measured total serum Ig levels (Figure 10E) and motor-activity (Table 8) in old Eps15-KO mice, but again failed to detect differences. These results suggest that the increase in MZ B cell number in Eps15-KO mice does not translate into evident functional alterations of the B-cell arm of the immune system.

**Table 8:** Normal motor activity in aged Eps15-KO mice.

Test	Eps15-WT (n = 9)	Eps15-KO (n = 10)
Locomotor activity		
# squares entered in 30″	28.0	33.2
Limb grasping		
0 = present (>30″)	6	7
1 = absent	3	3
mean score	0.3	0.3

Motoractivity was measured in aged (>20-month old) mice according to the modified SHIRPA protocol.

## Discussion

Here we report that, in mammals, Eps15 is involved in the regulation of lymphocyte homeostasis, most notably in the MZ B cell population. This is at variance with its established critical role in the nervous system in *C. elegans* and *D. melanogaster*. We demonstrate that Eps15 deficiency is associated with a reduction in circulating B cells and an increased capacity of mutant hematopoietic precursors to repopulate B, T and MZ B cell lineages but a reduced capacity to repopulate B1 B cells in the peritoneum.

The competitive BMT experiment uncovered a pleiotropic role of Eps15 in the immune system. Eps15 is an endocytic adaptor protein that interacts via several DPF motifs with AP-2, which is part of the hardware of clathrin-mediated endocytosis [46], [47], [48], [49], [50]. In addition, Eps15 possesses two UIM motifs that mediate binding to mono-ubiquitinated proteins; by virtue of this interaction Eps15 has also been implicated in non-clathrin mediated endocytosis and the sorting of ubiquitinated receptors [22], [51], [52]. Thus, Eps15 is likely involved in both clathrin and non-clathrin mediated endocytosis. A pleiotropic effect caused by the lack of Eps15 might, therefore, have been expected given the known importance of endocytic control in the regulation of signaling [53]. Eps15 is a ubiquitously expressed protein, whose absence has a differential impact in different cell types. This likely depends on the repertoire of receptors present in different cells, which results in a combinatorial effect on both signaling and on the developmental potential of any given cell type. Tissue-specific and/or inducible knockout mice for Eps15 will be needed to understand whether and, if so, how Eps15 regulates hematopoietic precursor proliferation, survival or migration.

The most notable phenotype, an increase in MZ B cell number, was observed under both challenged and steady-state conditions. There are several possible mechanisms that could explain altered MZ B cell homeostasis in Eps15-KO mice most of which we addressed experimentally. *i)* It is known that BCR surface levels, and signaling strength [18] direct MZ B cell development, although whether stronger BCR signaling favors Fo B or MZ B cell development is still a matter of debate [3], [45], [54], [55]. In particular it has been shown that transgenic mice over-expressing natural antibodies in increasing doses show increasing numbers of MZ B cells [19], [56]. We found that mutant MZ B cells show similar IgM, IgD, CD21 and CD19 surface levels compared with WT cells, ruling out that increased cell surface receptor levels drive the expansion of the MZ B cell population in Eps15-KO mice. *ii)* Similarly, it has been shown that the localization of the BCR to lipid rafts affects its signaling quality [57] and that different outcomes of BCR stimulation in immature versus mature B cells correlates with differing cholesterol levels in these cells [58] and with different localization to lipid rafts [59]. Given that Eps15, is an endocytic adaptor protein it might regulate recycling and/or trafficking of the BCR thereby affecting its signaling output. However, we observed no difference in BCR signaling in Eps15-KO and –WT splenic B cells, suggesting that BCR signaling is not altered in Eps15-KO mice. *iii)* Using conditional knockout mice and BMT experiments it was shown that Notch signaling is essential for MZ B cell development [13], [14], [15]. Two known Eps15 interactors are involved in regulating Notch signaling, Numb and Epsin. Numb is a well-established antagonist of Notch, originally identified in *D. melanogaster* [60]. Epsin (or liquid facet) was also originally identified in *D. melanogaster* where it participates in the internalization of the Notch ligand Delta [61], [62]. These data suggest that Eps15 might be involved in the regulation of Notch signaling. However, we did not observe any differences in the expression of Notch target genes [11] in MZ B cells from Eps15-KO mice, suggesting that altered Notch signaling is not involved in the MZ B cell phenotype in Eps15-KO. *iv)* One of the earliest events impinging on the developmental decision towards MZ B cell development is the repertoire of Ig variable region genes assembled during VDJ recombination at the Pro-B cell stage in the bone marrow. It has been shown that the “fetal-type” BCRs are enriched in MZ B cells indicating that a distinct VH repertoire preferentially guides MZ B cell development [63]. *v)* It has also been shown that both integrin and chemokine receptors are essential for proper homing of MZ B cells in the spleen [64], [65]. However, we did not observe any differences in the migration of Eps15-KO MZ B cells towards the stroma derived cytokine SDF-1 that might account for the increased MZB cell numbers observed in these mice. *vi)* It has been suggested that an expansion of the MZ B cell population might occur in situations of decreased generation of B cells from the bone marrow [66]). We observed normal production of B cells in the bone marrow and normal numbers of total splenic B cells, thus it is unlikely that MZ B cells are increased due to homeostatic expansion. *vii)* One interesting aspect of our findings is that MZ B cells are specifically affected, while transitional or pre-MZ B cell numbers appear normal. This would suggest that proliferation or apoptosis [67], [68] of MZ B cells themselves should be affected in Eps15-KO mice. However, we did not detect any differences neither in proliferation nor apoptosis of Eps15-KO cells *in vivo*. We did observe however, that the MZ B cell phenotype became more pronounced with age, suggesting that the phenotype develops slowly over time. Thus, if small changes in proliferation or apoptosis are responsible for the phenotype, it might be difficult to detect them experimentally.

The increase in MZ B cells in Eps15-KO mice did not influence lymphocyte reactivity in knockout mice in response to the pathogen Listeria and to the type-II T-cell independent antigen NP-FICOLL. Moreover, our data suggest that autoreactivity is not induced in Eps15-KO mice as a result of increased MZ B cells [44], [69].

Despite the importance of endocytosis in signaling, and in contrast to the strong phenotypes observed in *C. elegans* and *D. melanogaster*, we observed a relatively mild phenotype in Eps15-KO mice. This lack of an overt phenotype, especially in the nervous system, can likely be ascribed to functional redundancy between Eps15 and Eps15L1, which are both present in mammals. Eps15L1 is instead absent in both *C. elegans* and *D. melanogaster*. Eps15L1 is highly homologous to Eps15, displaying the same modular organization of 3 N-terminal EH domains, a central coiled-coil, and C-terminal DPF motifs and UIM domains [23], [70]. At present, it is not known whether specific binding partners exist for Eps15 or Eps15L1 and how cellular specificity might be dictated [29]. Given that, like Eps15, Eps15L1 is also expressed ubiquitously [71], one might speculate that in most cell types these two proteins have a redundant function. A function of Eps15 in the nervous system might be uncovered in a challenge situation or in the concomitant absence of Eps15L1. Nevertheless, the lack of an overt phenotype in the nervous system of Eps15-KO mice reinforces the idea that during evolution the endocytic network has gained both in complexity and in stability.

In conclusion in parallel with the evolution of a complex immune system, in mammals Eps15 has acquired a novel function in the regulation of lymphocyte homeostasis, most strikingly MZ B cell homeostasis. It remains to be determined whether this role is exclusive for Eps15 or whether it is (partially) shared with Eps15L1.

## Materials and Methods

### Mice

Mice were housed on a 12-h light/dark cycle in a SPF facility and had *ad libitum* access to water and food. Ly5.1 wild-type mice were purchased from Charles River. Eps15-KO mice were generated on a 129Sv/J background and then backcrossed for more than twelve generations onto a C57BL/6 background before being used for experiments. Most of the experiments were performed on mice derived from heterozygous crosses (i.e. Figures 1, 2, 3A–E, K–L, 4C–H, 8D, 10A, B, E). To reduce the number of mice for breeding the remaining experiments were performed on mice derived from homozygous crosses after no more than 3 generations.

Genotyping was performed on tail biopsies using standard PCR conditions at 60°C annealing with the following primers: Eps15-forward CTGTGGTTTCCAAATGTGTC, Eps15-reverse CGCCTCTTAATCATCATCATC and Neo-forward GATTACATAGTGAGTTCAAAGC.

### Ethics Statement

Experiments involving animals have been done in accordance with the Italian Laws (D.L.vo 116/92 and following additions), which enforces EU 86/609 Directive (Council Directive 86/609/EEC of 24 November 1986 on the approximation of laws, regulations and administrative provisions of the Member States regarding the protection of animals used for experimental and other scientific purposes). The authority responsible for ensuring that the provisions of EU 86/609 Directive are properly carried out in Italy is the Ministry of Health.

Accordingly to the regulatory requirements, our animal facility is fully authorized by the Italian Ministry of Health (DM N° 65/2007-A – July 4, 2007) and a Veterinarian Specialized in Laboratory Animal Science and Medicine) is responsible for the well-being of the experimental animals.

Currently, the Italian legislation does not require a specific ethical review process for all the experiments involving animals. A central (Government) review is required only for particular species (e.g., dogs, cats, and non-human primates) or for experiments performed without anesthesia or that will or may cause severe pain. In the other cases, only a notification of the experiments to the Ministry of Health is required. Accordingly, the project has been notified to the Ministry of Health (Project number 02/08).

### FACS Analysis

FACS analysis was performed on single cell suspensions after hypotonic red blood cell lysis, using the following antibodies: CD1d biotin and streptavidin PE, CD4 PE, CD8 FITC, CD21 PE, CD23 PE, IgD PE, CD5 PE, Gr-1 PE, B220 PE-Cy7, CD19 PE-Cy7, CD23 FITC, CD23 Alexa488, CD8 FITC, B220 APC, AA4.1 APC, CD38 APC, CD11c APC, CD45.1 APC Alexa750, CD45.2 PerCP Cy5.5, IgM APC, IGM Alexa 488, TCRbeta APC (ebioscience) and Fas PE, CD43 PE (Becton Dickinson). Samples were acquired on a FACSCanto II (Becton Dickinson) and were sorted on FACSAria. Data were analyzed with DIVA v 6.1.1 and Flojow 9.0/9.2 software.

### Immunofluorescence

Spleens were dissected, fixed overnight in 4% paraformaldehyde and 70 µm sections were cut on a Leica VT 100P vibratome. MZ B cells were identified as IgM^+^IgD^−^ cells, by staining with IgM-FITC and IgD-PE,. Confocal images were acquired with a Leica TCS SP2 AOBS Confocal microscope. Image analysis was performed using ImageJ.

### Immunoblotting

Immunoblotting was performed as previously described [72]. The following antibodies were employed: anti-Eps15 [24], anti-Eps15 (Sc-20, Santa Cruz), anti-vinculin (Sigma), anti- p-Akt and anti-Akt (Cell signaling).

### Internalization Assay

Internalization assays for EGF and transferrin was performed on primary mouse fibroblasts from the indicated genotypes, as previously described [22].

### Competitive BMT

For competitive BMT experiments, 5×10^6^ bone marrow cells were pooled from four 2–3 month old donor mice, which were obtained from heterozygous crosses. Mice had previously been backcrossed for more than 12 generations, however heterozygous crosses were performed to minimize the effect of the genetic background on the phenotype. Cells were injected into the tail vein of sub-lethally irradiated (7.5Gy) 3–4 month old recipient mice, which had received preventive antibiotic treatment (1 mg/ml ampicillin in drinking water) for 1 week.

### Behavioral Experiments

A modified SHIRPA protocol was employed to test for locomotor activity and grip strength as described by the European Mouse Phenotyping Resource of Standardized Screens (http://empress.har.mrc.ac.uk).

### Determination of Immunoglobulin Concentrations in the Serum of Mice

For the analysis of immunoglobulin isotypes within blood plasma samples we used a bead array-based Luminex system [73], which allows the simultaneous analysis of the different isotypes (IgG1, IgG2b, IgG3, IgA, and IgM) in a single sample. The level of each isotype was calculated over a standardcurve fitted with Four-Parameter-Logistic regression (Bio-Plex manager software, Bio-Rad).

### Listeria Infection

Infection experiments were performed with wild-type *Listeria monocytogenes* strain 10403 s. Brain Heart Infusion medium was inoculated with *Listeria* stock solution and incubated at 37°C until an OD600 of 0.05–0.1. After dilution with PBS mice were infected with 50.000 cfu in 100 µl by intravenous injection into the lateral tail vein. Although Listeria is not usually a blood born pathogen, using this mode,of adminstration would mean that it was presented as such. Three days after infection mice were sacrificed. Spleens and livers were homogenized. Serial dilutions of Triton-lysed organ homogenates (ranging from 1∶10 to 1∶1000) were plated on BHI agar plates. On the following day colony forming units (CFU) were quantified.

### Purification of Splenic B-cells

B cells were purified after RBC lysis by magnetic seperation from CD43+ and CD11b+ cells using biotinylated antibodies (BD and ebioscience, respectively) and anti-biotin microbeads (Miltenyi) over LS MACS columns (Mitenyi) according to the supplier’s protocol.

### Stimulation with Anti-IgM

Purified B cells were allowed to rest at 37°C and 5% CO_2_ in low adherence 24 well cell culture plates (Costar) in RPMI, 10% FCS, 50 µM beta-mercaptoethanol, 2 mM Glutamine and antibiotics at 5×10^6^ cells/500 µl for a minimum of 3 hrs prior to stimulation with 5 µg/ml of anti-mouse IgM (Jackson Immuno Research). After the indicated time points cells were spun down and immediately lysed in JS lysis buffer.

### Migration Assay

Migration assays were performed in 5 µm pore, 6 mm transwell inserts in 24- well plates. One million purified B cells in 100 µl were added to the upper well, while 600 µl medium, containing SDF-1 (Sigma) at the indicated concentrations, was added to the lower chamber. Cells were collected after 4 hrs and stained for surface markers prior to FACS analysis.

### Proliferation and Apoptosis Assays

Mice were injected intraperitoneally with 1 mg BrdU in 0.1 ml PBS. After 4 hrs mice were sacrificed and spleens collected for analysis. Staining for BrdU using anti-BrdU FITC (ebioscience) was performed after DNase (Sigma) treatment using the CytoFix/CytoPerm kit (BD Bioscience) prior to staining for surface markers. Staining for active Caspase-3 was performed by incubating cells with FITC-DEVDK-FMK (BioVision), a cell permeable Caspase inhibitor for 1 hr at 37°C and 5% CO_2_ prior to fixation and staining for surface markers.

### QPCR

QPCR was performed as described previously [74].
