# Correction: The Endocytic Adaptor Eps15 Controls Marginal Zone B Cell Numbers

**DOI:** 10.1371/annotation/9c1c760a-8477-499d-a194-5ed87ffa6c6a

**Published:** 2013-05-17

**Authors:** Benedetta Pozzi, Stefania Amodio, Caterina Lucano, Anna Sciullo, Simona Ronzoni, Daniela Castelletti, Thure Adler, Irina Treise, Ingrid Holmberg Betsholtz, Birgit Rathkolb, Dirk H. Busch, Eckhard Wolf, Helmut Fuchs, Valérie Gailus-Durner, Martin Hrabě de Angelis, Christer Betsholtz, Stefano Casola, Pier Paolo Di Fiore, Nina Offenhäuser

There were errors in two affiliations and in the Funding statement of the article.

Affiliations 3 and 6 should read, respectively:

3 German Mouse Clinic, Institute of Experimental Genetics, Helmholtz Zentrum München, Munich-Neuherberg, Germany,

6 Institute for Molecular Animal Breeding and Biotechnology, Ludwig-Maximilians-Universität München, Munich, Germany,

The Funding statement should read: This work was funded by grants from AIRC (Associazione Italiana per la Ricerca sul Cancro), Fondazione Monzino and Fondazione Ferrari to PPDF, and by grants from the German Federal Ministry of Education and Research (NGFN-Plus grant numbers 01GS0850 to MHdA, 01GS0851 to EW, and 01GS0852 to DB; Infrafrontier 01KX1012 to the GMC) and the European Union (EUMODIC LSHG-2006–037188). SC is supported by the Italian Foundation for Cancer Research (FIRC). The funders had no role in study design, data collection and analysis, decision to publish, or preparation of the manuscript. 

